# Efficacy and safety of SGLT2 inhibitors in the treatment of maturity-onset diabetes of the young (MODY): a case report and literature review

**DOI:** 10.1007/s42000-025-00632-8

**Published:** 2025-02-04

**Authors:** Michelle Bombonato, Guglielmo Beccuti, Andrea Benso, Beatrice Giannone, Silvana Bertaina, Fabio Broglio, Fabio Bioletto

**Affiliations:** Division of Endocrinology, Diabetes and Metabolism, Department of Medical Sciences, University of Turin, Corso Dogliotti 14, 10126 Turin, Italy

**Keywords:** Monogenic diabetes, Maturity-onset diabetes of the young, MODY, SGLT2 inhibitors, Empagliflozin

## Abstract

**Background:**

Sulfonylureas constitute the standard therapy for patients with HNF1A-MODY (maturity-onset diabetes of the young) but are characterized by an increased risk of hypoglycemia. While SGLT2 inhibitors (SGLT2i) may potentially represent a useful therapeutic option, data from the literature are scant.

**Case presentation:**

We report the case of a young woman affected by HNF1A-MODY who was successfully and safely treated with an SGLT2i in addition to sulfonylurea. After SGLT2i initiation, an improvement in the patient’s glycemic control was observed and was maintained over time. No adverse effects were noted and, in particular, no increase in ketonemia or ketonuria occurred.

**Conclusions:**

The use of SGLT2i under controlled circumstances may represent a useful therapeutic option in patients with HNF1A-MODY.

**Supplementary Information:**

The online version contains supplementary material available at 10.1007/s42000-025-00632-8.

## Introduction

Maturity onset diabetes of the young (MODY) is a heterogeneous group of monogenic diabetes [1] characterized by differences in disease severity, clinical manifestations, therapeutic options, and prognosis. Diagnosis of MODY is still challenging, it being estimated that nearly 70 to 80% of patients are not correctly diagnosed [1, 2]. This not only leads to missed family screening but also ineffective management given that, in MODY, a correct diagnosis is essential for the tailoring of treatment.

SGLT2 inhibitors (SGLT2i) are a class of antihyperglycemic drugs which have demonstrated significant extra-glycemic benefits, particularly in terms of cardiovascular and renal protection [3] in type 2 diabetes. Their potential use in other forms of diabetes is thus a topic that has aroused much enthusiasm and interest. However, their efficacy and safety in patients with monogenic diabetes are far from being fully understood. To date, dedicated clinical trials on this topic are lacking and the available evidence mostly relies on case reports. An updated systematic review of the available evidence is provided in the Supplementary Materials. Herein we describe the case of a patient affected by HNF1A-MODY treated effectively and safely with SGLT2i as an add-on therapy to sulfonylurea.

## Case presentation

We describe the case of a young woman who was first diagnosed with diabetes after a short course of steroid treatment for otitis media in 2009 at the age of 17. Since random glucose during infection was found to be high on two occasions (respectively, 286 and 418 mg/dL), HbA1c was measured, which was 8.7% (72 mmol/mol). Her weight was normal (BMI 20 kg/m^2^) and she had a strong family history of diabetes (father, paternal grandmother, maternal uncle, and maternal grandfather). Her other medical history was unremarkable. An autoimmune form of diabetes was excluded by measuring islet cell antibodies (ICA), glutamic acid decarboxylase 65 (GAD65) antibodies, and insulinoma-associated protein 2 (IA-2) antibodies, which were all negative. Stimulated C-peptide levels were normal (C-peptide after glucagon test 3ng/mL). In light of these presenting features (young age, negative autoantibodies panel, family history, and normal BMI), our patient was referred for genetic testing for heritable forms of diabetes. The genetic analysis revealed a heterozygous variant (c.283 A > T) in the promoter of the HNF1A gene which was considered pathogenetic for HNF1A-MODY according to the clinical presentation. Genetic testing was negative for HNF4A-MODY and GCK-MODY-associated mutations. After genetic confirmation, treatment adjustments were made and metformin was first substituted with a low dose of glimepiride and, subsequently, with gliclazide and acarbose. Genetic testing was offered both to her father and to her siblings, confirming the presence of a mutation responsible for MODY3 in the father and her sister (who developed hyperglycemia some years thereafter). In contrast, her brother was not a carrier of the mutation. During follow-up, the patient continued treatment with gliclazide modified release 60 mg/day as monotherapy and her glycemic control has been optimal for years. She experienced only a single episode of severe symptomatic hypoglycemia (47 mg/dL) after nausea and vomiting, which needed intravenous glucose administration for resolution.

In the previous year, her glycemic control started to get slightly worse, with her HbA1c rising to 7.4% (57 mmol/mol) in April, 2023. Therefore, we agreed with the patient to attempt initiation of an SGLT2i (empagliflozin 10 mg/day) as an add-on therapy to gliclazide, which was continued at the same dose. This strategy was preferred to an up-titration of the gliclazide dose in order to minimize the risk of hypoglycemia. Moreover, it was preferred over other drug classes for several other reasons, including its excellent tolerability profile, its simplicity in administration (oral route), and the potential additional independent benefits in the prevention of cardiovascular and renal complications. Upon SGLT2i therapy initiation, the patient was instructed to undertake abundant hydration and careful hygienic care and was given a sick day protocol.

After both 6 months (October, 2023) and 12 months of treatment (April, 2024), optimal glycemic control was achieved (HbA1c 6.5% [47 mmol/mol] and 5.8% [40 mmol/mol], respectively, with no hypoglycemia). The patient’s weight decreased by 2 kg after 6 months of treatment and by an additional 5 kg after 1 year. The therapy was well tolerated with no adverse effects; in particular, the patient did not report any genitourinary infection during treatment. The estimated glomerular filtration rate, calculated using the CKD-EPI formula, remained unchanged before and after empagliflozin initiation (106 mL/min and 111 mL/min, respectively); the same held true for hematocrit (46 and 43%, respectively). As a further check on the safety of the treatment, a fingerstick ketonemia level was measured in our clinic and was within the normal range (0.2 mmol/L) at both 6 and 12 months after empagliflozin initiation. Urinalysis was also negative for ketones and demonstrated glycosuria as expected during SGLT2i therapy. Table 1 summarizes the changes over time in key clinical and biochemical parameters since the initiation of SGLT2i therapy.

**Table 1:** Changes over time in key clinical and biochemical parameters since the initiation of SGLT2i therapy. *Abbreviations*: BMI, body Mass Index; eGFR, estimated glomerular filtration rate

Parameter	Baseline	6 months	12 months
HbA1c (%)	7.4	6.5	5.8
HbA1c (mmol/mol)	57	47	40
Fasting plasma glucose (mg/dL)	130	108	76
Weight (kg)	60	58	53
BMI (kg/m^2^)	22.9	22.1	20.2
Fingerstick ketonemia (mmol/L)	-	0.2	0.2
Ketonuria (urine dipstick)	-	Negative	Negative
eGFR (mL/min/1.73m^2^) ^a^	106	111	89
Haematocrit (%)	46	43	43

^a^ Estimated according to the CKD-EPI formula

## Discussion

Herein we describe the safe and effective use of an SGLT2i in a patient affected by HNF1A-MODY with insufficient glycemic control when treated with gliclazide as monotherapy.

HNF1A diabetes is the most common type of MODY requiring treatment and the prevalent form in northern Europe [2, 4]. The HNF1A gene codes for a transcription factor expressed in many tissues, such as the pancreas, the liver, and the kidneys [2, 4]. Within the pancreas, HNF1A acts as a weak transactivator of the insulin gene in beta cells, thus leading to defective insulin secretion when mutated [4], while in the kidneys, HNF1A physiologically regulates SGLT2 channel expression and activity in the renal tubules [4–6]. Pathogenic variants in the HNF1A gene may thus result in a low renal threshold for glucose, causing glycosuria with low levels of hyperglycemia even before overt diabetes develops, which can be an early marker in the natural history of the disease [2, 4].

Patients with this form of diabetes respond well to low-dose sulfonylureas [2, 4], at least in the first few years after diagnosis. Insulin deficiency can occur later during disease history and insulin therapy is thereafter required [4, 7]. Given that these patients already exhibit naturally decreased renal SGLT2 activity, treatment with SGLT2i may seem redundant from a pathophysiological perspective. In practice, however, the use of SGLT2i could add to further promoting glucose excretion, thereby providing additional benefits in glycemic control.

So far, two other cases of SGLT2i use in patients with HNF1A-MODY have been described in the literature [5, 8]. In both cases, the outcomes in terms of glycemic control were reported only in the short term, with no availability of data about changes in HbA1c. Moreover, one case raised concerns about the safety of the treatment as the patient developed ketosis after being started on SGLT2i; on the other hand, it must be acknowledged that the development of this side effect took place under some very specific circumstances since the patient was following a very-low-carbohydrate ketogenic diet with the aim of achieving weight loss. In our case, by contrast, the effectiveness of the treatment was demonstrated over a longer period, with a documented improvement in HbA1c of 1.6% over a time span of 12 months. Moreover, in our patient, no safety concerns arose as the treatment was well tolerated and no evidence of ketosis was documented.

In our patient, empagliflozin was added several years after diagnosis of MODY when sulfonylurea treatment was found to be no longer sufficient for maintenance of optimal glycemic control. Thus, the choice to add an SGLT2i to gliclazide averted further deterioration of glycemic control and potentially postponed initiation of insulin treatment. No data are available on the hypothesis that SGLT2i could have delayed initiating insulin by preserving beta cell mass and function in patients affected by MODY, even though in both animal models and individuals with type 2 diabetes or prediabetes, SGLT2i as a class seem to exert a role in ameliorating insulin sensitivity and secretion [9]. The correct timing for introduction of gliflozins in the therapeutic plan of subjects with MODY (i.e., at diagnosis, taking advantage of a theoretical protective action on beta cells, or, should the need arise, after secondary failure of sulfonylurea) is at present far from being fully understood: it would be of the highest significance to explore this topic to a greater extent.

It is well known that patients with HNF1A-MODY have the same risk of developing microvascular and macrovascular complications as those with type 1 diabetes and type 2 diabetes [1, 4, 10], especially in cases of poor glycemic control [4]. In recent years, advancements in diabetes treatment have led to the introduction of new medication classes (e.g., SGLT2i and GLP1-receptor agonist, GLP-1 RA) that are not only efficacious in terms of glycemic control but also offer additional independent benefits in the prevention of cardiovascular and renal complications [3, 11]. Notably, although such benefits were initially demonstrated in patients with type 2 diabetes, they have subsequently also been demonstrated in populations of patients without diabetes, i.e., in patients with heart failure [12] and chronic kidney disease [13] (for SGLT2i), or in patients with obesity (for GLP1-RA) [14, 15], thereby suggesting that their benefits may be, at least partially, independent of the specific pathophysiology of type 2 diabetes. Given these premises, the use of SGLT2i and GLP1-RA could be a tempting therapeutic option for the management of various forms of monogenic diabetes [7].

Clearly, the information that can be drawn from this particular case is limited. This is an observational study based on a single patient and, therefore, it is difficult to generalize. Moreover, the follow-up time is 12 months, with no data about the safety profile and the efficacy on glycemic control in the longer term. Finally, the possible additional benefits in terms of delayed insulin initiation, cardiorenal protection, and overall prevention of diabetes complications remain speculative and yet to be demonstrated.

In conclusion, to our knowledge, this is the first case report in the literature pointing to the safety and efficacy of SGLT2i in HNF1A-MODY beyond the short term. Nevertheless, in order to generalize these findings, clinical trials are needed to comprehensively evaluate the possible benefits and harms of this drug class in MODY patients. In the absence of compelling evidence in this sense, when prescribing this class of drugs in patients with monogenic diabetes, careful patient selection and strict follow-up are advisable to closely monitor the efficacy, safety, and tolerability of the treatment.

## Electronic supplementary material

Below is the link to the electronic supplementary material.

Supplementary Material 1
