# Supplementary material for: Efficacy and safety of SGLT2 inhibitors in the treatment of maturity-onset diabetes of the young (MODY): a case report and literature review
Source: Hormones (Athens). 2025 Feb 4;24(2):495–8. doi: 10.1007/s42000-025-00632-8 (PMC12339573; doi:10.1007/s42000-025-00632-8)
Supplement: Supplementary file 1 — Supplementary Material 1 [file 42000_2025_632_MOESM1_ESM.pdf]

SUPPLEMENTARY MATERIAL

Literature review

We performed a comprehensive screening of PubMed/Medline library to identify relevant and pertinent studies about the use of SGLT2i in patients with monogenic diabetes. The complete search string used was the following: ("sodium glucose co-transporter 2" OR "sodium-glucose co-transporter 2" OR "sodium glucose co-transporter-2" OR "sodium-glucose co-transporter-2" OR "sodium glucose cotransporter 2" OR "sodium-glucose cotransporter 2" OR "sodium glucose cotransporter-2" OR "sodium-glucose cotransporter-2" OR "SGLT2" OR "SGLT2i" OR "SGLT2-i" OR "SGLT2 inhibitor" OR "SGLT2 inhibitors" OR "SGLT2-inhibitor" OR "SGLT2-inhibitors" OR "empagliflozin" OR "dapagliflozin" OR "canagliflozin" OR "ertugliflozin") AND ("MODY" OR "maturity onset diabetes of the young" OR "maturity-onset diabetes of the young" OR "monogenic diabetes"). The search was updated through May 3rd 2024 and identified 22 results.

Among the 22 results of the search string, 7 studies reported original data about the effects of the administration of SGLT2i in human patients with monogenic diabetes [S1–S8]. More specifically, one was a pathophysiological study [S8], which aimed to compare the glycosuric response to a single dose of dapagliflozin in patients with HNF1A-MODY, GCK-MODY, and type 2 diabetes; the other seven were case reports[S1–S7] describing the outcomes of the chronic treatment with SGLT2i in different types of monogenic diabetes.

In the pathophysiological study [S8], the authors enrolled 14 HNF1A-MODY, 19 GCK-MODY, and 12 type 2 diabetes patients. All studied individuals received a single morning dose of 10 mg of dapagliflozin added to their current anti-diabetic therapy; response to dapagliflozin was assessed by evaluating the change induced in urinary glucose-to-creatinine ratio. The main result of the study was that dapagliflozin was able to induce higher glycosuria in patients with HNF1A-MODY and GCK-MODY compared to those with type 2 diabetes, without differences between the two MODY forms. The main limitation was the lack of long-term data on the potential effects of chronic treatment, as the authors themselves acknowledged.

With regard to the case reports, the use of SGLT2i was reported in various forms of monogenic diabetes, as summarised in Supplementary Table 1.

The first description of their use was published by Ovsyannikova et al in 2016 [S1]. The authors described the case of a 27-year-old man who developed diabetes and soon after diagnosis was complicated by pre-proliferative retinopathy and macular oedema, arterial hypertension, and distal sensory neuropathy. The patient was initially treated with insulin but had recurrent hypoglycaemic episodes even with low insulin doses. A genetic diagnosis of MODY due to mutation in the ATP-binding cassette, subfamily C, member 8 (ABCC8) gene was made in the patient and his mother, and the therapy was modified accordingly (insulin discontinued, gliclazide started). Because of persistent glucose excursions during this treatment, together with a tendency to fasting hypoglycaemia that prevented gliclazide dose escalation, the therapy was implemented by adding dapagliflozin 10 mg/day. In this way, good glycaemic control was finally achieved, demonstrated both by HbA1c and continuous glucose monitoring profiles. Concerning safety, the therapy was apparently well-tolerated, and no presence of ketones in the urine was detected.

Some years later, Brodosi et al. [S2] reported the case of a 48-year-old man who was first classified as being affected by type 2 diabetes when he was 25 years old and had been treated with insulin basal-bolus therapy and metformin since then. When he first came to the attention of the authors, prandial insulin therapy was stopped, and dapagliflozin 10 mg/day was added to therapy. The autoimmune aetiology of diabetes was again excluded, and genetic testing was performed. Treatment with SGLT2i was interrupted shortly after initiation due to abdominal pain that spontaneously resolved after suspension. At last, a mutation in the neurogenic differentiation factor 1 (NEUROD1) gene was found and gliclazide was introduced optimising and simplifying glycaemic control.

The same year, Yeung et al. [S3] described three cases of patients with mitochondrial diabetes due to mitochondrially encoded tRNA leucine 1 (MT-TL1) mutation treated with SGLT2i. One case was that of a 51-year-old man who, after initial unsuccessful management with metformin, was transitioned to empagliflozin 25 mg/day in association with sitagliptin and gliclazide, but could not achieve an adequate glycaemic control (HbA1c 9.0%, 75 mmol/mol); later, subcutaneous semaglutide was initiated as monotherapy, with the achievement of HbA1c levels < 7% (53 mmol/mol). The second case was that

of a young 32-year-old woman, who was initially treated with metformin and gliclazide without obtaining an adequate glycaemic control (HbA1c 8.0%, 64 mmol/mol); later, she was transitioned to empagliflozin 25 mg/day as monotherapy, with an improvement in HbA1c (7.0%, 53 mmol/mol) and a good tolerance of the treatment. Finally, the case of a 72-year-old woman is reported, who could not achieve adequate glycaemic control with glyburide and sitagliptin (HbA1c 9.5%, 80 mmol/mol); the addition of basal insulin was trialled, but failed due to recurrent hypoglycaemia even when administered at low doses; when empagliflozin 25 mg/day was started in association with sitagliptin, glyburide was discontinued, and the glycaemic control significantly improved (HbA1c 8.0%, 64 mmol/mol), without reported adverse events.

Still, in 2021, the use of SGLT2i for the treatment of two patients with HNF1A-MODY was reported by Sriravindrarajah et al. [S4] and by Phan et al. [S5]. In the first work [S4], the authors described the case of a 30-year-old woman who was hospitalised due to osteomyelitis and septic arthritis in the setting of a chronic diabetic foot ulcer. During hospitalisation, poor glycaemic control was recorded despite treatment with supramaximal doses of metformin and gliclazide. Therefore, empagliflozin 10 mg/day was successfully added to the therapy, with a prompt improvement of glycaemic control and the possibility to reduce the doses of metformin and gliclazide. Regrettably, no follow-up data are provided in the longer term.

In the other report, Phan et al. [S5] described the case of a 25-year-old woman with HNF1A monogenic diabetes, in which dapagliflozin 10 mg/day was initiated in substitution of her previous treatment with metformin and insulin degludec at low dose (2 IU/day). At the time, the patient was following a very low-carbohydrate ketogenic diet (VLCKD, carbohydrate intake < 10 g/day) with the aim of weight loss, which she also continued after the therapeutic switch to SGLT2i. The therapy with SGLT2i was able to ensure good glycaemic control at self-monitoring, but after nearly two months of treatment, the patient incidentally discovered positive ketonaemia at fingerstick (3.8 mmol/L), despite the absence of overt symptoms. The patient was euglycaemic, and no evidence of acidosis was found (pH 7.41) when she arrived at the emergency department. Ketosis was resolved by insulin administration. In light of this episode, the previous therapy with metformin and basal insulin at very low dose was restored.

In 2023, Suzuki et al. [S6] described the use of SGLT2i as an adjunctive treatment in the setting of HNF4A-MODY. More specifically, they reported the case of a 24-year-old man who could not achieve satisfactory glycaemic control (HbA1c 8%, 64 mmol/mol) during treatment with gliclazide at high doses (160 mg/day). The addition of empagliflozin 10 mg/day to his treatment plan led to a significant improvement in glycaemic control, with the achievement of HbA1c levels < 7% (53 mmol/mol), good tolerability and no relevant side effects reported.

Finally, in 2024, Zhao et al. [S7] reported the case of a man diagnosed with GCK-MODY in his forties. In the years following diagnosis, due to persistently suboptimal HbA1c levels, the patient was treated sequentially with several hypoglycemic agents, including an SGLT2i. More specifically, after therapeutic trials with metformin, sitagliptin, pioglitazone and acarbose, empagliflozin (dose not specified) was started. HbA1c did not change over a treatment period of 6 months, and therefore treatment was discontinued and replaced with a new drug, dorzagliatin, with benefits on glycaemic control. Nevertheless, empagliflozin was well tolerated with no adverse events and, in particular, no ketosis developed.

Supplementary References

S1. Ovsyannikova AK, Rymar OD, Shakhtshneider EV, et al (2016) ABCC8-Related Maturity-Onset Diabetes of the Young (MODY12): Clinical Features and Treatment Perspective. Diabetes Ther 7:591–600. https://doi.org/10.1007/s13300-016-0192-9

S2. Brodosi L, Baracco B, Mantovani V, Pironi L (2021) NEUROD1 mutation in an Italian patient with maturity onset diabetes of the young 6: a case report. BMC Endocr Disord 21:202. https://doi.org/10.1186/s12902-021-00864-w

S3. Yeung RO, Al Jundi M, Gubbi S, et al (2021) Management of mitochondrial diabetes in the era of novel therapies. J Diabetes Complications 35:107584. https://doi.org/10.1016/j.jdiacomp.2020.107584

S4. Sriravindrarajah A, Fernandes A, Wu T, Hocking S (2021) The use of SGLT2 inhibitors in achieving glycaemic control in maturity-onset diabetes of the young type 3. Endocrinol Diabetes Metab Case Rep 2021:21–0102, EDM-21–0102. https://doi.org/10.1530/EDM-21-0102

S5. Phan F, Bourron O, Laroche S, et al (2021) Euglycaemic diabetic ketosis decompensation under dapagliflozin in a patient with MODY3. Diabetes Metab 47:101248. https://doi.org/10.1016/j.diabet.2021.101248

S6. Suzuki S, Kokumai T, Furuya A, Takahashi S (2023) SGLT2i as a Useful Adjunctive Medication for HNF4A-MODY. Diabetes Care 46:e74–e75. https://doi.org/10.2337/dc22-1573

S7. Zhao Y, Ma Y, Ba T, et al (2024) Hypoglycemic Response to Dorzagliatin in a Patient With GCK-MODY. Diabetes Care dc232417. https://doi.org/10.2337/dc23-2417

S8. Hohendorff J, Szopa M, Skupien J, et al (2017) A single dose of dapagliflozin, an SGLT-2 inhibitor, induces higher glycosuria in GCK- and HNF1A-MODY than in type 2 diabetes mellitus. Endocrine 57:272–279. https://doi.org/10.1007/s12020-017-1341-2

First author, year	Mutated gene	Patients' characteristics	SGLT2i and dosage	Efficacy	Tolerability and safety
Ovsyannikova, 2016 [S1]	ABCC8	27-year-old man	Dapagliflozin 10 mg/day	Improvement of HbA1c and CGM data	Apparently well-tolerated, no evidence of ketonuria
Brodosi, 2021 [S2]	NEUROD1	48-year-old man	Dapagliflozin 10 mg/day	N/A	Discontinued due to abdominal pain
Yeung, 2021 [S3]	MT-TL1 (mitochondrial)	51-year-old man	Empagliflozin 25 mg/day	Inadequate glycaemic control and HbA1c	Apparently well-tolerated
		32-year-old woman	Empagliflozin 25 mg/day	Improvement of HbA1c	Well-tolerated
		72-year-old woman	Empagliflozin 25 mg/day	Improvement of HbA1c	Apparently well-tolerated
Sriravindrarajah, 2021 [S4]	HNF1A	30-year-old woman	Empagliflozin 10 mg/day	Improvement of glycaemic control in the short term	Apparently well-tolerated in the short term
Phan, 2021 [S5]	HNF1A	25-year-old woman	Dapagliflozin 10 mg/day	Good glycaemic control in the short term	Euglycaemic ketosis without acidosis during VLCKD
Suzuki, 2023 [S6]	HNF4A	24-year-old man	Empagliflozin 10 mg/day	Improvement of HbA1c	Apparently well-tolerated
Zhao, 2024 [S7]	GCK	45-year-old man	Empagliflozin (dose not specified)	No improvement of HbA1c	Apparently well-tolerated, no evidence of ketosis

Supplementary Table 1. Summary of patients' characteristics and outcomes of the available case reports in the literature. *Abbreviations*: ABCC8, ATP-binding cassette, subfamily C, member 8; CGM, continuous glucose monitoring; GCK, glucokinase; HNF1A, hepatocyte nuclear factor-1-alpha; HNF4A, hepatocyte nuclear factor-4-alpha; MT-TL1, mitochondrially encoded tRNA leucine 1; NEUROD1, neurogenic differentiation factor 1; N/A, not available; SGLT2i, SGLT2 inhibitor; VLCKD, very low-carbohydrate ketogenic diet.
